# Towards Development of an Anti-Vampire Bat Vaccine for Rabies Management: Inoculation of Vampire Bat Saliva Induces Immune-Mediated Resistance

**DOI:** 10.3390/v13030515

**Published:** 2021-03-20

**Authors:** Horacio A. Delpietro, Roberto G. Russo, Charles E. Rupprecht, Gabriela L. Delpietro

**Affiliations:** 1 Servicio Nacional de Sanidad y Calidad Agroalimentaria (SENASA), Padre Serrano 1116, 3300 Posadas, Argentina; reviroseco@hotmail.com (R.G.R.); gabi.delpietro@gmail.com (G.L.D.); 2 LYSSA LLC, 309 Pirkle Ferry Rd., Cumming, GA 30040, USA; charleserupprechtii@gmail.com

**Keywords:** anticoagulant, blood, control, lyssavirus, rabies, saliva, vampire bat, zoonosis

## Abstract

The common vampire bat (*Desmodus rotundus*) is a hematophagous species responsible for paralytic rabies and bite damage that affects livestock, humans and wildlife from Mexico to Argentina. Current measures to control vampires, based upon coumarin-derived poisons, are not used extensively due in part to the high cost of application, risks for bats that share roosts with vampires and residual environmental contamination. Observations that vampire bat bites may induce resistance in livestock against vampire bat salivary anticoagulants encourage research into novel vaccine-based alternatives particularly focused upon increasing livestock resistance to vampire salivary components. We evaluated the action of vampire bat saliva-Freund’s incomplete adjuvant administered to sheep with anticoagulant responses induced by repeated vampire bites in a control group and examined characteristics of vampire bat salivary secretion. We observed that injections induced a response against vampire bat salivary anticoagulants stronger than by repeated vampire bat bites. Based upon these preliminary findings, we hypothesize the utility of developing a control technique based on induction of an immunologically mediated resistance against vampire bat anticoagulants and rabies virus via dual delivery of appropriate host and pathogen antigens. Fundamental characteristics of host biology favor alternative strategies than simple culling by poisons for practical, economical, and ecologically relevant management of vampire populations within a One Health context.

## 1. Introduction

**Figure 1:**
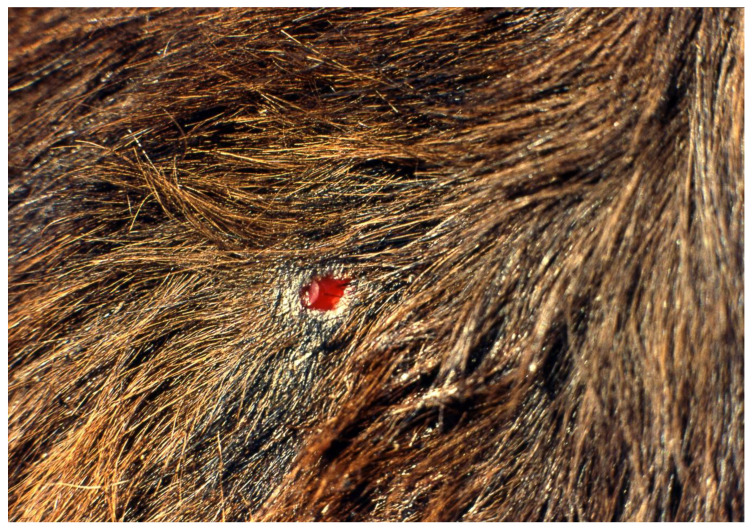
A typical lesion after a feeding bite of the common vampire bat on cattle. In a single bite, the vampire bat removes a piece of skin ~6 mm, exposing the subcutaneous tissue of the prey, and licks the blood. The anticoagulants in the bat’s saliva facilitate the maintenance of bleeding during feeding.

The common vampire bat (*Desmodus rotundus*) is a strict hematophagous species that ranges from northern Mexico to Uruguay and to central Chile and Argentina [1,2]. Today, vampires abound, feeding mainly on the blood of livestock, poultry, wildlife and humans [3,4,5,6]. Vampire bat hematophagy is unique among mammals. This adaptation is inherent to anatomical and physiological characteristics of the bat’s digestive system. Briefly, vampire bats have specialized canine and incisor teeth that allow removal of a piece of skin from prey in a single bite (Figure 1), exposing the subcutaneous tissue (sometimes deep within the muscle) from which they lick the flowing blood of the host [1,6,7,8,9,10,11,12].

**Figure 2:**
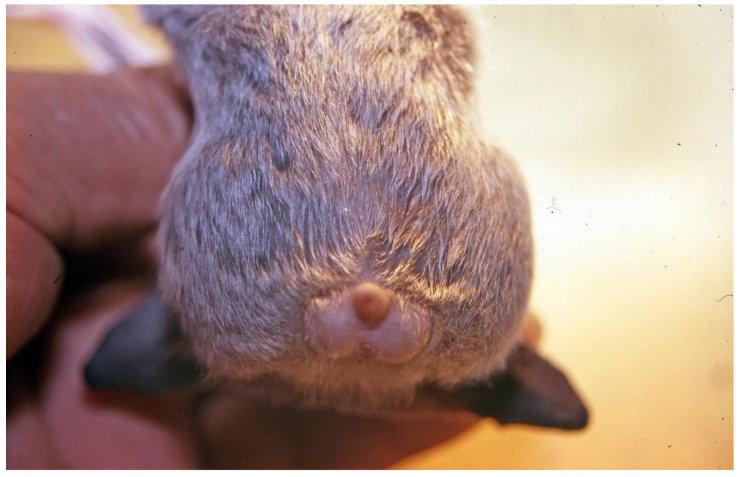
A male vampire bat captured while feeding in the wild, with a distended abdomen, not yet totally filled with blood. Salivary anticoagulants facilitate the maintenance of the blood flow during ingestion and in a liquid state within the digestive tract of the bat. Blood of prey, with strong resistance to vampire bat anticoagulants induced by an appropriate delivery, could coagulate within the digestive tract of the bat, making both digestion and elimination of excess water difficult.

Vampire bat salivary glands secrete saliva containing anticoagulants and fibrinolytic enzymes that prevent blood clotting, both during ingestion as well as inside the gastrointestinal tract during the processes of digestion and elimination of excess water [13,14,15,16,17,18,19,20,21]. The tubular cecum and extensibility of the gastrointestinal tract allow for rapid ingestion and digestion of a large blood volume (Figure 2). During each feeding bout that lasts 20–30 min, vampire bats ingest a blood quantity close to their own weight (roughly 30 mL), and in less than 120 min, defecation begins [6,7,8,9,22,23].

**Figure 3:**
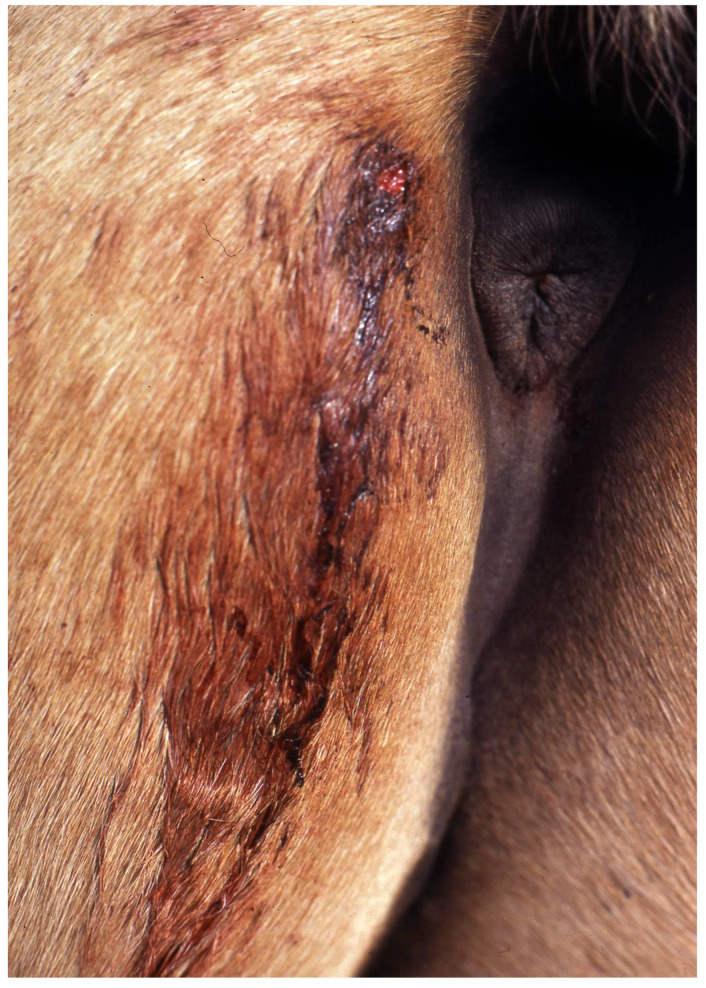
Residual hemorrhage in a horse. Blood continues to flow from the bite after the vampire bat finishes feeding due to the residual effect of its salivary anticoagulants.

Elimination of excess water through urination begins 2 to 4 min after the blood intake begins and continues for more than two hours [10,11,12]. Vampire bats feed nightly [10,11,12]. Searching for suitable prey expends a high energetic cost [24,25]. After feeding, the wound on the prey continues to bleed (Figure 3) due to the persistent effect of salivary anticoagulants [6,7,8,9,26].

Vampire bats are the primary reservoir of the rabies virus in tropical and subtropical areas of the Americas. Annually, “paralytic rabies” kills tens of thousands of livestock, dozens of humans and an indeterminate quantity of wildlife [4,5,6,26,27,28,29,30,31,32]. Additionally, wounds and blood loss due to vampire bat bites cause serious damage (Figure 3), including anemia, infections, physical pain and psychological suffering in humans; anemia, loss of vision, loss of weight and productivity and predisposition to infections and myiasis in livestock; and mortality from excessive bleeding in poultry, wild birds and other small prey [33,34,35,36,37]. While the risk of rabies acquisition may be minimized by vaccination of humans, domestic animals or wildlife, such prevention does not impact the burden associated with the outcome from overt vampire bat feeding upon prey.

Current measures to control vampire bats based on the use of coumarin-derived poisons [38,39,40] are generally used only at a local level because their high application cost makes it difficult or impossible to maintain treatment over time in large areas such as provinces or countries [41,42]. Furthermore, due to the toxic risk for non-target species of bats and other mammals that share roosts with vampires [4,5,6,43,44,45,46,47,48], such use is recommended to be reduced to the minimum possible and to seek other control measures [4,5,27,28,41,49].

Observations that repeated vampire bat bites induce in cattle immunologically mediated resistance against vampire salivary anticoagulants [50], and of the preference of vampire bats for biting those animals apparently less resistant to their salivary anticoagulants, such as young individuals and newcomers from vampire bat-free sites [50,51,52,53], encourage applied research for control alternatives to present methods, particularly those focused on increasing resistance of livestock against vampire salivary anticoagulants. In this study, we analyzed aspects of the antigenic behavior of vampire bat saliva inoculated parenterally in sheep. Our main objectives were to: determine whether subcutaneous inoculation of vampire bat saliva-Freund’s incomplete adjuvant could induce an immunologically mediated resistance against vampire bat anticoagulants; evaluate the response of inoculated sheep compared with that induced by repeated vampire bat bites in a control group; compare the interference capacity against vampire bat salivary anticoagulants of sheep sera before and after inoculation with bat saliva-Freund’s incomplete adjuvant, and observe characteristics of vampire bat salivary secretion during saliva collection.

## 2. Materials and Methods

### 2.1. Abbreviations

CT: clotting time (min); VSA: vampire salivary anticoagulants; mix 1: a sample of sheep blood mixed with vampire saliva in a 29:1 ratio, used to observe its CT when evaluating the resistance of sheep against VSA (see below Section 2.7); mix 2: a sheep serum sample mixed with vampire saliva, and blood from the reference sheep in a 6:1:20 ratio, used to observe its CT when evaluating the neutralizing capacity of sera before and after sheep were injected with vampire saliva-Freund’s incomplete adjuvant (see below Section 2.8).

### 2.2. Sheep

We used 24 Hampshire Down ewes raised in our Laboratory of 12/24 months of age and a mean weight of 46 kg. Sheep had an inbreeding rate >80%, daughters from the same father, and descendant by maternal lines from the same grandfather and great-grandfather, and they were immunologically naive with respect to vampire bat saliva since they had not been bitten previously by vampire bats nor injected with vampire bat saliva. Sheep were divided into groups A, B and C, composed of 12, 11 and 1 sheep each, respectively. Taking advantage that sheep left their corral one-by-one crossing a narrow working chute, on 8 July 2019, we formed the experimental groups, proceeding as follows: group C was the first sheep that left the corral; group A consisted of those sheep from even-numbered departures: 2°,4°…24°; and group B with those from odd-numbered departures: 3°,5°…23°, which were identified by numbered ear tags. Sheep of group A were used in the assay of inoculation of vampire bat saliva, those of group B in the assay of exposure to vampire bat bites and the single individual of group C was used as a blood source in the tests to evaluate the neutralizing capacity of sheep serum against VSA. All procedures used in the experiments with sheep were approved by the Ethics Committee of the Consejo Profesional de Médicos Veterinarios de la Provincia de Misiones, Argentina.

### 2.3. Vampire Bats

We used 62 adult common vampire bats captured, quarantined, and held in captivity during the study, as described [50]. Briefly, they were caught during 3–8 March 2019, in areas where there were no reported livestock rabies outbreaks during the previous 2 years. After a 70-day quarantine in which they remained healthy, and rabies virus was not isolated from 2 saliva samplings made 26 and 60 days after capture, the bats were considered appropriate for use in the study. The bats were divided into 2 groups: 48 vampire bats were used for the extraction of saliva, and 14 of them were used for feeding on sheep. Both the collection in the field, as well as the management in captivity of vampire bats, were made under Provincial and Federal permits and followed the guidelines approved by the American Society of Mammalogists for the use of wild mammals in research [54].

### 2.4. Extraction, Preservation, and Manipulation of Vampire Bat Saliva

We collected vampire bat saliva during May–July (Table 1), proceeding as follows: a bat was restrained, and to stimulate salivation, their mouth was rinsed 3–4 times with 0.5 mL of 0.4% pilocarpine solution, administered with a plastic syringe without a needle. Afterward, the bat was hung head down for 10 min, and the saliva secretion was collected in a Petri dish placed on ice under the bat. The quantity of saliva secreted by 8–11 bats (about 7–9 mL) was pipetted from the Petri dish and combined in a tube for the elimination of cells and other impurities by centrifugation. The supernatant was kept in another labeled tube and maintained at −30 °C. When the quantity of saliva was accumulated, the tubes were thawed, and saliva was placed together in a dish on wet ice, where it was mixed carefully for 15 min and redistributed in tubes that were kept at −30 °C until use. In this way, we were able to use saliva of the same origin and with the same handling (i.e., 2 freezing periods and 2 thaws).

### 2.5. Inoculation of Sheep with Vampire Bat Saliva

Vampire bat saliva was injected mixed in equal parts with incomplete Freund’s adjuvant (Sigma-Aldrich, #F5506). The mixture was prepared 15/20 min before its application by mixing the components with a syringe until obtaining an emulsion. Inoculations were performed subcutaneously in group A sheep, as follows: 2 mL during July and 1 mL during August and during October 2019 (Table 1).

### 2.6. Exposure of Sheep to Vampire Bat Bites

Between 15 July and 12 November 2019, sheep of group B were exposed to vampire bat bites (Table 1). In each exposure session, 5–6 sheep were placed into a room where 12 vampires were kept from dusk to 23 h. Exposure sessions were held every 2–3 days. After exposure, bites suffered by sheep were counted, and a topical insecticide was applied to avoid myiasis.

### 2.7. Evaluation of the Resistance of Sheep against Vampire Bat Salivary Anticoagulants

We observed the CT values of sheep blood mixed with vampire bat saliva in a 29:1 ratio (mix 1), proceeding as follows: we placed 0.1 mL of vampire bat saliva in a tube and added 2.9 mL of the freshly drawn blood sample, which was mixed by inverting and rotating the tube, which was then placed vertically at 38 °C, tilting every 3–4 min to observe the CT; as the relationship between these parameters were negative, high values of mix 1 CT indicated scarce resistance of sheep against VSA and vice versa. We observed the CT of mix 1 to evaluate and compare the resistance against VSA in the sheep of group A, before and after inoculation with saliva and in those of group B before and after exposing them to vampire bat bites (Table 2).

### 2.8. Evaluation of the Interference Capacity of sheep against Vampire Bat Salivary Anticoagulants

To evaluate this aspect, we observed the CT values of serum mixed with vampire saliva and blood from the reference sheep (mix 2), proceeding as follows: we placed in a tube 0.6 mL of sheep serum sample and 0.1 mL of vampire bat saliva, mixing and incubating at 38 °C for 30 min; then, to enable this mixture for coagulation and CT observation, we added 2 mL of freshly drawn blood from the reference sheep, mixing and observing the CT, as previously described. Although we did not know if the blood of reference sheep used in these tests to achieve the coagulation could have influenced the CT results, this does not invalidate the observations, since when using blood from the same sheep, the influence would have been the same in all tests as an in-house control. We used these tests to evaluate and compare the interfering capacity of serum in group A sheep before and after they were injected with vampire bat saliva and incomplete Freund’s adjuvant (Table 1 and Table 3).

### 2.9. Statistics

We used paired *t*-tests to compare the CT values of mix 1, when we evaluated the resistance against VSA in sheep of groups A and B, before and after they were injected or exposed to vampire bat bites, respectively (Table 2); and of mix 2 when we compared the interfering capacity against VSA of sera of group A sheep before and after they were injected (Table 3). We used a 2-sample t-test to compare in sheep groups A and B the resistance acquired against VSA after they were injected or exposed to vampire bat bites, respectively (Table 2). In essence, our basic objective was to compare the resistance against VSA induced by vampire bat saliva–incomplete Freund’s adjuvant injections and by vampire bites. In all statistical tests, the *p*-values < 0.05 were considered statistically significant.

## 3. Results

### 3.1. Extraction of Saliva

We obtained 91 mL of saliva in 123 extraction sessions of 10 min of duration each, with an average secretion per vampire/extraction session of 0.7 mL.

### 3.2. Exposure of Sheep to Vampire Bat Feeding Bites

During 4 months of observation (Table 1), sheep received between 55 and 61 bites each (a level of predation pressure somewhat higher than that expected in naturally exposed Argentine sheep).

### 3.3. Resistance of Sheep against VSA

We observed a significant decrease in the CT average of mix 1 from 37.7 to 21 min (t = 8.1, *p* = 0.000006) in group A sheep, after the injections of bat saliva–incomplete Freund’s adjuvant, and from 38.6 to 28.3 min (t = 5.3, *p* = 0.00004) in group B sheep, after exposure to vampire bat bites (Table 2), indicating that both saliva injections and vampire bat bites induce immunologically mediated resistance against VSA. We also observed that the decrease in the CT average of mix 1 was significantly greater in sheep of group A than in those of group B, t = −3.58, *p* < 0.005 (Table 2), indicating that injections of vampire bat saliva–incomplete Freund’s adjuvant induced immunologically mediated resistance against VSA stronger than that induced by vampire bat feeding-bites.

### 3.4. Interference Capacity of Ser against VSA.

The observation of a decrease from 22.8 to 11.3 min (*t* = 19.9, *p* << 0.0001) in the CT average of mix 2 (Table 3) shows the increase in the interference capacity of serum against VSA by the injections.

## 4. Discussion

Our findings showed that administration of vampire bat saliva–incomplete Freund’s adjuvant-induced interfering responses against VSA stronger than those induced by repeated vampire bat bites. We hypothesize that neutralizing antibodies are an important component (perhaps the main effector?) of this response. Observations during vampire bat saliva extractions indicated that the mean secretion per vampire bat per 10-min extraction session was 0.7 mL, an amount close to 1.8% of the vampire bat’s weight (40 g), suggesting that during each feeding bout in the wild, lasting 20 to 30 min [6,7,8,9], vampire bats secrete approximately 1.5 mL of saliva.

Clearly, vampire bats can secrete relatively large amounts of saliva rapidly, indicating the importance of preventing blood coagulation. Considering the results of the present study, we suggest the utility of developing an alternative method for the control of vampire bats, based upon induction of a strong immune response of livestock against VSA, by cloning and expression of appropriate “anti-vampire” salivary vaccine antigens. In theory, such a biologic could promote blood clotting in immunized cattle (due to its acquired resistance against VSA), making it difficult for the bat to ingest an adequate amount of blood, as well as its proper digestion and elimination of excess water. Assumedly, it would be difficult for vampire bats to survive feeding upon highly VSA-resistant prey, even more so considering the severe demands and limitations that hematophagy imposes. During each feeding bout, vampire bats need to ingest an amount of blood approximating their own weight [1,10,11,12]. Vampire bats need to feed productively nightly; otherwise, they may not survive more than 48–72 h of fasting [10,11,12]. Moreover, they must feed relatively quickly (i.e., within 20/30 min) because while feeding, they are an easy prey target for predators [6,55]. Searching for suitable prey has a high energetic cost since this activity may take up to 2.4 h [24]. Each hour spent foraging requires an estimated 1.1 additional Kcal., or 2.7 mL of blood [25]. Furthermore, this enterprise is even more difficult for female bats due to the energy demands of pregnancy, lactation, and feeding of the young by regurgitation, in that they require an even higher blood intake than males [1,6,9].

The development of an effective form of immune control could provide a key tool in vampire bat management since by not using poisons, and by routine use to free-ranging animals, such a strategy would lack the limitations of current control measures [4,5,6,43,44,45,46,47,48]. Suitable poisons that meet regulatory requirements are increasingly difficult to acquire. Utilization requires individual intra-rumen inoculation, treatment of bite lesions of cattle with paste or application to vampire bats directly. The capture of vampires is quite time-consuming and occurs over multiple nights. Training is required for the proper use and the identification of vampire bats to distinguish them from other bats. In fact, such a new endeavor would be innocuous for other species of bats, as well as for other wildlife, including those that prey upon vampire bats or share their roosts [6,55]. Likewise, such a biologic would be safer to handle by operators, and its application would be simpler and at a presumably lower cost, as it may be applied by local farmers themselves, as occurs routinely with most vaccines for livestock in rural areas. However, the generation of health economic data is beyond the scope of the present work. Such preliminary observations, as reported here, only suggest one of the preliminary practical steps in the search for a transdisciplinary alternative to the control of vampire bats. Obviously, achieving such a broader objective will require a longer study path, as is generally the case in the development of new biologics, particularly of those against non-microbial pathogens, such as vaccines against ectoparasites, such as ticks [56,57,58,59,60,61]. However, we believe that the potential of this and related methodology hold promise in terms of safety for other species and downstream environmental concerns, as well as ease of use and potentially lower-cost applications, which may justify such efforts, given the expanding burden throughout the region. The utilization of recombinant rabies viruses as expression vectors may provide one strategy as a candidate for dual vaccination against both the pathogen and the predator [62]. Such a control technique would protect livestock directly and negatively impact vampire bats, taking into account the close relationship between the size of vampire populations with the intensity, duration and frequency of rabies virus outbreaks [3,6,41]. As rabies remains a core vaccine, ideally, all domestic animals at risk of exposure, including livestock, should be vaccinated, especially in areas where vampire bat rabies is enzootic. Future research may result in the development of a new safe and effective concomitant rabies and anti-vampire bat vaccine for relevant prevention and control in a One Health context.

## Figures and Tables

**Table 1:** Tasks performed during the study in experimental sheep. Groups: A (injected with vampire bat saliva) and B (exposed to vampire bat bites).

Date	Group	Task
10 July	A	Blood sampling for resistance testing against VSA
11 July	B	Blood sampling for resistance testing against VSA
12 July	A	Blood sampling for extraction of sera
13 July	A	Tests of the neutralizing capacity of sera against VSA
15 July	B	Start of exposure sessions to vampire bat bites
17 July	A	First injection of vampire bat saliva–incomplete Freund’s adjuvant
20 August	A	Second injection of vampire bat saliva–incomplete Freund’s adjuvant
21 October	A	Third injection of vampire bat saliva–incomplete Freund’s adjuvant
12 November	B	Completion of exposure sessions to vampire bat bites
2 December	A	Blood sampling for resistance testing against VSA
3 December	B	Blood sampling for resistance testing against VSA
5 December	A	Blood sampling for extraction of sera
6 December	A	Tests of the neutralizing capacity of sera against VSA

**Table 2:** Observation of the clotting time (min) of * mix 1 to evaluate resistance against ** VSA in sheep of groups A and B after they were administered vampire bat saliva–incomplete Freund’s adjuvant or exposed to vampire bat bites, respectively.

Clotting Time (min) of mix 1, Statistical Data	Group A of 12 Sheep Injected with Vampire Saliva-Incomplete Freund’s Adjuvant		Group B of 11 Sheep Exposed to Vampire Bat Bites	
	before Shots	after Shots	before Bites	after Bites
Minimum	32	13	34	22
Maximum	47	30	47	36
Median	36	22	37	29
Mean	37.7	21	38.6	28.3
Skewness/kurtosis	1/−0.1	0.5/−0.2	1/0–0.4	0.2/0.4
Conf. int. of mean	34.6–40.7	17.9–24	35.6–41.6	25.6–30.9
Standard deviation	4.8	4.8	4.4	4
Sum	452	252	425	311
Comparison by paired *t*-test	*t* = 8.1, *p* = 0.000006		*t* = 5.3, *p* = 0.00004	

* mix 1: a mixture of a sample of sheep blood freshly drawn and vampire saliva in a ratio 29:1, respectively (see Section 2.7 in the text) ** VSA: vampire salivary anticoagulants.

**Table 3:** Observation of the clotting time (min) of * mix 2 to evaluate the interference capacity against ** VSA of the sera of group A sheep before and after administration of vampire bat saliva–incomplete Freund’s adjuvant.

Clotting Time (min), Statistical Data	Before Inoculation	After Inoculation
Minimum	19	9
Maximum	29	15
Median	22.5	11
Mean	22.8	11.3
Skewness/kurtosis	0.9/0.4	0.6/−0.7
Conf. int. of mean	21/24.7	10/12.5
Standard deviation	2.9	2.0
Sum	274	135
Comparison by paired *t*-test: *t* = 19.9, *p* << 0.0001		

* mix 2: mixture of the sheep serum sample, vampire bat saliva and blood of the reference sheep (see Section 2.8 in the text). ** VSA: vampire salivary anticoagulants.

## Data Availability

Not applicable.

## References

[1] Greenhall A.M., Joermann G., Schmidt U. (1983). Desmodus rotundus. Mamm. Species.

[2] Koopman K.F., Greenhall A.M., Schmidt U. (1988). Systematics and distribution. Natural history of Vampire Bats.

[3] Delpietro H., Marchevsky N., Simonetti E. (1992). Relative population densities and predation of the common vampire bat (Desmodus rotundus) in natural and cattle-raising areas in north-east Argentina. Prev. Veter. Med..

[4] Johnson N., Aréchiga-Ceballos N., Aguilar-Setien A. (2014). Vampire Bat Rabies: Ecology, Epidemiology and Control. Viruses.

[5] Streicker D.G., Allgeier J.E. (2016). Foraging choices of vampire bats in diverse landscapes: Potential implications for land-use change and disease transmission. J. Appl. Ecol..

[6] Delpietro H.A., Russo R.G., Carter G.G., Lord R.D., Delpietro G.L. (2017). Reproductive seasonality, sex ratio and philopatry in Argentina’s common vampire bats. R. Soc. Open Sci..

[7] Paradiso J.L., Goodwin G.G., Greenhall A.M. (1961). A Review of the Bats of Trinidad and Tobago: Descriptions, Rabies Infection, and Ecology. J. Mammal..

[8] Greenhall A.M. (1972). The biting and feeding habits of the Vampire bat, *Desmodus rotundus*. J. Zoöl..

[9] Greenhall A.M., Schmidt U., Lopez-Forment W. (1969). Field observations on the mode of attack of the vampire bat *Desmodus ro-tundus* in Mexico. An. Inst. Biol. Univ. Aut. México.

[10] Wimsatt W.A. (1969). Transient Behavior, Nocturnal Activity Patterns, and Feeding Efficiency of Vampire Bats (Desmodus rotundus) under Natural Conditions. J. Mammal..

[11] Wimsatt W.A., Guerriere A. (1962). Observations on the Feeding Capacities and Excretory Functions of Captive Vampire Bats. J. Mammal..

[12] McFarland W.N., Wimsatt W.A. (1969). Renal function and its relation to the ecology of the vampire bat, Desmodus rotundus. Comp. Biochem. Physiol..

[13] Disanto P.E. (1960). Anatomy and histochemistry of the salivary glands of the vampire bat, desmodus rotundus murinus. J. Morphol..

[14] Hawkey C. (1967). Inhibitor of Platelet Aggregation Present in Saliva of the Vampire Bat Desmodus rotundus. Br. J. Haematol..

[15] Gardell S.J., Duong L.T., Diehl R.E., York J.D., Hare T.R., Register R.B., Jacobs J.W., Dixon R.A., Friedman P.A. (1989). Isolation, characterization, and cDNA cloning of a vampire bat salivary plasminogen activator. J. Biol. Chem..

[16] Krätzschmar J., Haendler B., Langer G., Boidol W., Bringmann P., Alagon A., Donner P., Schleuning W.D. (1991). The plasminogen activator family from the salivary gland of the vampire bat Desmodus rotundus: Cloning and expression. Gene.

[17] Krätzschmar J., Haendler B., Bringmann P., Dinter H., Hess H., Donner P., Schleuning W.D. (1992). High-level secretion of the four salivary plasminogen activators from the vampire bat *Desmodus rotundus* by stably transfected baby hamster kidney cells. Gene.

[18] Fernandez A.Z., Tablante A., Bartoli F., Beguin S., Hemker H., Apitz-Castro R. (1998). Expression of biological activity of draculin, the anticoagulant factor from vampire bat saliva, is strictly dependent on the appropriate glycosylation of the native molecule. Biochim. Biophys. Acta (BBA) Gen. Subj..

[19] Fernandez A.Z., Tablante A., Beguín S., Hemker H., Apitz-Castro R. (1999). Draculin, the anticoagulant factor in vampire bat saliva, is a tight-binding, noncompetitive inhibitor of activated factor X. Biochim. Biophys. Acta (BBA) Protein Struct. Mol. Enzym..

[20] Low D.H., Sunagar K., Undheim E.A., Ali S.A., Alagon A.C., Ruder T. (2013). Dracula’s children: Molecular evolution of vampire bat venom. J. Proteomics..

[21] Ware F.L., Luck M.R. (2017). Evolution of salivary secretions in haematophagous animals. Biosci. Horizons Int. J. Stud. Res..

[22] Mitchell G.C., Tigner J.R. (1970). The route of the ingested blood in the common vampire bat (*Desmodus rotundus*). J. Mammal..

[23] Rouk C.S., Glass B.P. (1970). Comparative Gastric Histology of Five North and Central American Bats. J. Mammal..

[24] Young A.M. (1971). Foraging of vampire bats *Desmodus rotundus* in Atlantic wet lowland Costa Rica. Rev. Biol. Trop..

[25] Breidenstein C.P. (1982). Digestion and Assimilation of Bovine Blood by a Vampire Bat (*Desmodus rotundus*). J. Mammal..

[26] Baer G.M., Baer G.M. (1975). The biology and control of vampire bats. The Natural History of Rabies.

[27] World Health Organization (2004). WHO Expert Consultation on Rabies: First Report.

[28] World Health Organization (2013). WHO Expert Consultation on Rabies: Second Report.

[29] Rupprecht C.E., Turmelle A., Kuzmin I.V. (2015). A perspective on lyssavirus emergence nd perpetuation. Curr Opin Virol..

[30] Rupprecht C.E., Kuzmin I.V. (2015). Why we can prevent, control and possibly treat, but will not eradicate rabies. Future Virol..

[31] Da Rosa E.S., Kotait I., Barbosa T.F., Carrieri M.L., Brandão P.E., Pinheiro A.S., Begot A.L., Wada M.Y., De Oliveira R.C., Grisard E.C. (2006). Bat-transmitted human rabies outbreaks, Brazilian Amazon. Emerg. Infect. Diseases.

[32] Delpietro H.A., Lord R., Russo R.G., Gury-Dhomen F. (2009). Observations of sylvatic rabies in Northern Argentina during out-breaks of paralytic cattle rabies transmitted by vampire bats (*Desmodus rotundus*). J. Wildl. Dis..

[33] Kverno N.B., Mitchell G.C. (1976). Los murciélgos vampiros y la producción pecuaria en. Am. Lat. Rev. Mund. Zoot..

[34] Thompson R.D., Elias D.J., Mitchell G.C. (1977). Effects of Vampire Bat Control on Bovine Milk Production. J. Wildl. Manag..

[35] Greenhall A.M., Greenhall A.M., Schmidt U. (1988). Feeding behavior. Natural History of Vampire Bats.

[36] Crespo J., Vanella J., Blood B., De Carlo J.M. (1961). Observaciones ecológicas del vampiro Desmodus rotundus rotundus (Geoffroy) en el norte de Córdoba. Rev. Mus. Arg. Cien. Nat. B Rivadavia.

[37] Delpietro H.A., Russo R.G., Schwieters H.H.G. (1999). Observaciones sobre el ataque del vampiro común (*Desmodus rotundus*) al ganado en el norte de Argentina. Rev. Med. Vet..

[38] Linhart S.B., Flores Crespo R., Mitchell G.C. (1972). Control de murciélagos vampiros por medio de un anticoagulante. Bol. Oficina Sanit. Panam..

[39] Schmidt U., Schmidt C., Lopez-Forment W., Flores Crespo R. (1978). Rückfunde beringter Vampirfledermaüse (*Desmodus rotundus*) in Mexico. Z. Säugetierkunde.

[40] Flores Crespo R., Ibarra V.F., De Anda D.L. (1976). Vampirinip II: Un producto utilizable en tres métodos para el combate del mur-ciélago hematófago. Téc. Pecu. Méx..

[41] Delpietro H.A., Nader A.J. (1989). Rabies of herbivores transmitted by vampire bats in north-eastern Argentina. Rev. Sci. Tech. Off. Int. Epiz..

[42] Seetahal J.F.R., Vokaty A., Carrington C.V., Adesiyun A.A., Mahabir R., Hinds A.Q.J., Rupprecht C.E. (2017). The History of Rabies in Trinidad: Epidemiology and Control Measures. Trop. Med. Infect. Dis..

[43] Greenhall A.M., Greenhall A.M., Artois M., Fekadu M. (1993). Ecology and bionomics of vampire bats in Latin America. Bats and Rabies.

[44] Walker S., Medellín M.R.A., Aguirre L.A., Mann A., Ochoa J.R. (2001). Conservation progress in Latin America. Bat. Mag..

[45] Mayen F. (2003). Haematophagous Bats in Brazil, Their Role in Rabies Transmission, Impact on Public Health, Livestock Industry and Alternatives to an Indiscriminate Reduction of Bat Population. J. Veter Med. Ser. B.

[46] Asprilla-Aguilar A.A., Mantilla-Meluk H., Jiménez Ortega A.M. (2007). Analysis of the non-hematophagous bat species captured within the plan of eradication of Desmodus rotundus (E. Geoffroy, 1810) in the Colombian Biogeographic Chocó. Rev. Inst. Univ. Tecnol. Chocó Invest. Biod. Des..

[47] Oprea M., Aguliar L.M.S., Wilson D.E. (2009). Anoura caudifer (*Chiroptera: Phyllostomidae*). Mamm. Species.

[48] Aguiar L.M.S., Brito D., Machado R.B. (2010). Do Current Vampire Bat (*Desmodus rotundus*) Population Control Practices Pose a Threat to Dekeyser’s Nectar Bat’s (*Lonchophylla dekeyseri*) Long-Term Persistence in the Cerrado?. Acta Chiropterologica.

[49] Rocha F., Dias R.A. (2020). The common vampire bat Desmodus rotundus (*Chiroptera: Phyllostomidae*) and the transmission of the rabies virus to livestock: A contact network approach and recommendations for surveillance and control. Prev. Veter. Med..

[50] Delpietro H.A., Russo R.G. (2009). Acquired resistance to saliva anticoagulants by prey previously fed upon by vampire bats (*Des-modus rotundus*): Evidence for immune response. J. Mammal..

[51] Arellano C.S., Sureau P., Greenhall A.M. (1971). Preferencia de la predación del vampiro en relación a la edad y la raza del ganado y la época del año. Téc. Pec. Méx..

[52] Dalquest W.W. (1955). Natural History of the Vampire Bats of Eastern Mexico. Am. Midl. Nat..

[53] Acosta y Lara E.F. (1950). Quirópteros del Uruguay. Com. Zool. Mus. Hist. Nat. Montev..

[54] Gannon W.L., Sikes R.S. (2007). Guidelines of the American Society of Mammalogists for the Use of Wild Mammals in Research. J. Mammal..

[55] Delpietro H., Konolsaisen F., Marchevsky N., Russo G. (1994). Domestic cat predation on vampire bats (*Desmodus rotundus*) while foraging on goats, pigs, cows and human beings. Appl. Anim. Behav. Sci..

[56] Willadsen P., Bird P., Cobon G.S., Hungerford J. (1995). Commercialization of a recombinant vaccine against *Boophilus microplus*. Parasitology.

[57] Willadsen P. (2004). Anti-tick vaccines. Parasitology.

[58] Trimnell A.R., Hails R.S., Nuttall P.A. (2002). Dual action ectoparasite vaccine targeting ‘exposed’ and ‘concealed’ antigens. Vaccine.

[59] Nuttall P.A., Trimnell A.R., Kazimirova M., Labuda M. (2006). Exposed and concealed antigens as vaccine targets for controlling ticks and tick-borne diseases. Parasite Immunol..

[60] De la Fuente J., Contreras M. (2015). Tick vaccines: Current status and future directions. Expert Rev. Vaccines.

[61] Rego R.O.M., Trentelman J.J.A., Anguita J., Nijhof A.M., Sprong H., Klempa B., Ehajdusek O., Tomás-Cortázar J., Azagi T., Strnad M. (2019). Counterattacking the tick bite: Towards a rational design of anti-tick vaccines targeting pathogen transmission. Parasites Vectors.

[62] Scher G., Schnell M.J. (2020). Rhabdoviruses as vectors for vaccines and therapeutics. Curr. Opin. Virol..

